# Associations between urban metrics and mortality rates in England

**DOI:** 10.1186/s12940-016-0106-3

**Published:** 2016-03-08

**Authors:** Daniela Fecht, Lea Fortunato, David Morley, Anna L. Hansell, John Gulliver

**Affiliations:** UK Small Area Health Statistics Unit, MRC-PHE Centre for Environment and Health, Imperial College London, London, W2 1PG UK; Imperial College Healthcare NHS Trust, London, W2 1NY UK

## Abstract

**Background:**

Seventy-five percent of the population in Europe live in urban areas and analysing the effects of urban form on the health of the urban population is of great public health interest. Not much is known, however, on the effects of urban form on the health of city dwellers. This study uses a novel approach to investigate whether associations exist between different measures of urban form and mortality risks in cities in England.

**Methods:**

We conducted an ecological, cross-sectional study for urban areas in England with more than 100,000 residents (*n* = 50) and included all registered premature deaths (<65 years) between 1^st^ January 2002 and 31^st^ December 2009. To describe and categorise urban form we quantified the distribution and density of population, land cover and transport networks and measures of geographical characteristics. We used Poisson regression models to examine associations between the measures of urban form and age-standardised risks of deaths from all causes, cardiovascular disease, and traffic accidents after adjustment for socioeconomic status and smoking. Analysis was stratified by gender to explore differential associations between females and males.

**Results:**

There were a total of 200,200 premature deaths during the study period (Females: 37 %; Males: 63 %). Transport network patterns were associated with overall and cardiovascular mortality rates in cities. We saw 12 % higher mortality risk after adjustment in cities with high junction density compared to cities with low density [Females: RR 1.12 (95 % CI 1.10 – 1.15); Males: RR 1.12 (95 % CI 1.10–1.14)]; the risk was slightly higher for cardiovascular mortality [Females: RR 1.16 (95 % CI 1.10 – 1.22); Males: RR 1.12 (95 % CI 1.09 – 1.16)]. Associations between mortality and population patterns were of similar magnitude [Females: RR 1.10 (95 % CI 1.09 – 1.13); Males: RR 1.09 (95 % CI 1.07–1.10)]; associations between mortality and land cover patterns were inconclusive.

**Conclusions:**

We found an association between transport patterns and risk of premature mortality. Associations between urban form and mortality observed in this study suggest that characteristics of city structure might have negative effects on the overall health of urban communities. Future urban planning and regeneration strategies can benefit from such knowledge to promote a healthy living environment for an increasing urban population.

**Electronic supplementary material:**

The online version of this article (doi:10.1186/s12940-016-0106-3) contains supplementary material, which is available to authorized users.

## Background

Seventy-five percent of the population in Europe live in cities. Analysing the effects of urbanisation and urban influences on the health and wellbeing of the urban population is therefore of great public health interest [1]. Urban areas are characterised by dynamic and complex patterns in spatial structure and function [2, 3]. Understanding these complexities and their interactions with external factors, has, for several decades, been the focus of many urban scientists, cutting across disciplinary lines. Urban and transport planners, as well as social science disciplines, such as geography, sociology, economics and political sciences, try to unravel the complex nature of the city and its consequences [4]. Epidemiologists likewise have for centuries analysed the spatial distribution of disease in urban areas. In fact, the first studies in epidemiology all centred around the major conurbations of the time [5, 6].

**Fig. 1:**
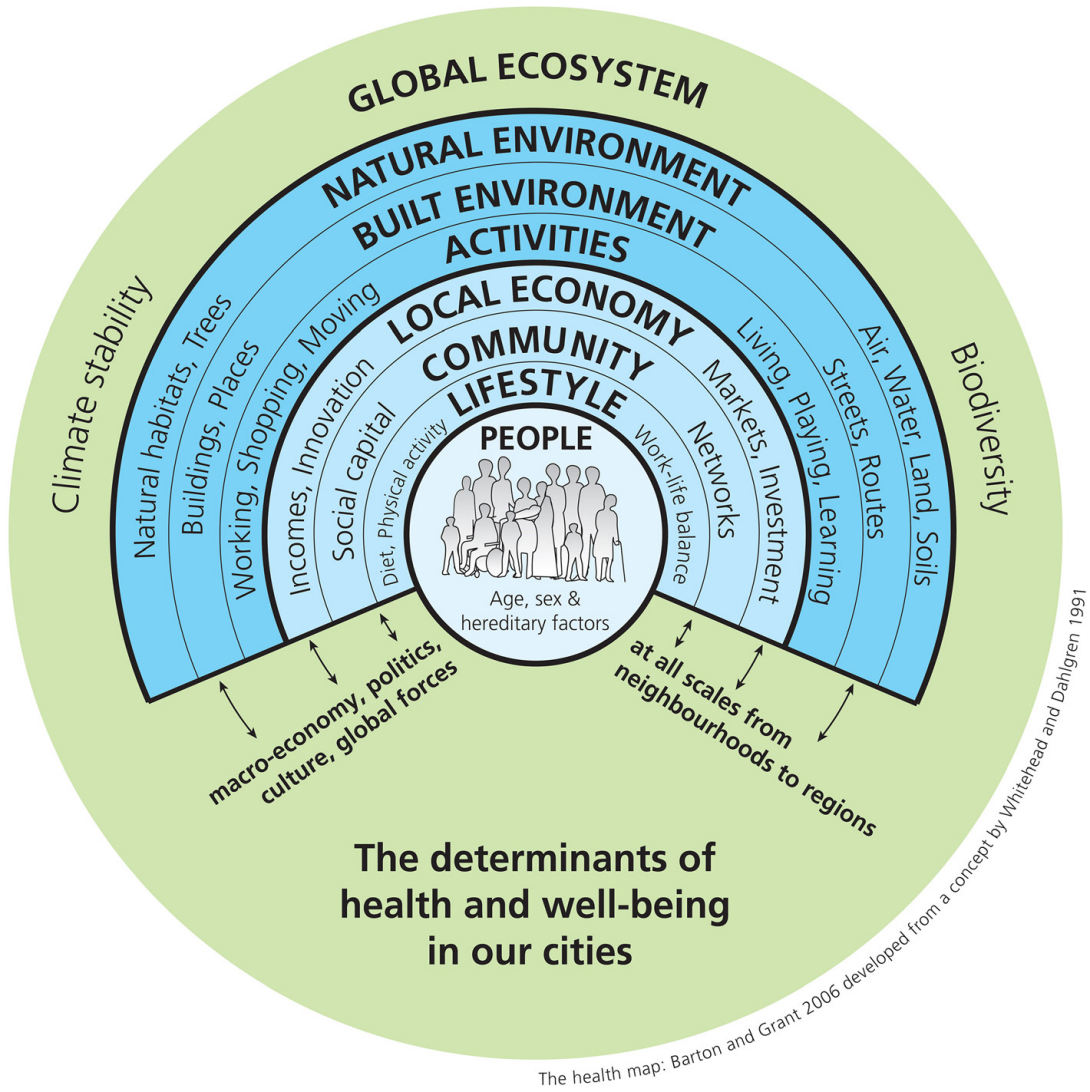
The determinants of health in our cities [48, 49] reproduced under The Health Map Creative Commons License

Cities impact on the health of its residents both negatively and positively [7]. Disease and mortality rates in the urban population are, besides genetic and lifestyle causes, influenced by multiple social and environmental factors that form a complex system of causality as illustrated by Fig. 1. The spatial variability of air pollution exposure, with its well established cardiovascular and respiratory health effects [8], for example, is mostly a result of the spatial distribution of specific land uses (e.g. industrial land, transport related uses) and general meteorological conditions. These, in turn, can be influenced by the terrain, street design and urban layout. Urban air quality is also influenced by the amount of parks and open spaces in a city which again might have an effect on urban climate and its associated health effects [9] as well as on the physical activity levels of urban residents which are related to the risk of obesity, diabetes and cardiovascular disease [10, 11]. In addition, historical artefacts such as industrial heritage, traditional pockets of deprivation and the temporal city development have an influence on the wellbeing of urban residents [12]. All these factors combine to make the built urban environment an important determinant of the health of the general population.

Research on the associations between city living and urban health in recent years has focused on how specific urban characteristics potentially promote the health of urban residents. Recent studies, for example, have identified associations between access to green spaces and lower rates of mortality [13] and reduced stress levels [14]. Others have established a relationship between the walkability of cities (in terms of residential densities, land use mix and street layout) and its multiple positive impacts on public health including an increase in physical activity, reduction in air pollution emissions and decrease in road traffic accidents [15].

Most of these studies have focused on how local characteristics in the residential neighbourhood impact on health. Some urban characteristics, in particular related to physical features and the urban structure might act at a broader, city-wide scale. To our knowledge there are no comprehensive studies to date that investigated whether urban characteristics at the city level are factors that influence the health of the urban population. That is, is the layout of a city indicative of the overall health of its inhabitants? This study tries to answer this question by using a cross-sectional ecological approach, to compare associations between physical features, urban structure and mortality rates in English cities.

## Methods

We investigated the relationship between all-cause and cause-specific mortality and characteristics of urban form for cities in England, using a cross-sectional, ecological study design.

### Unit of analysis

Cities were our units of analysis which we defined as all continuous urban areas with a population ≥ 100,000 (*n* = 50). Population numbers were derived from the 2001 census population for urban areas produced by the Office for National Statistics (ONS) [16]. ONS defines urban areas based on the extent of urban development on Ordnance Survey (OS) maps (at least 20 ha) and by a minimum population of 1,500 people in the 2001 census. Transportation features and urban green land are included in this approach, playing fields and golf courses are excluded if not surrounded by built-up areas. ONS assigned Census Output Areas (COA), a small area census dissemination unit with an average of 300 residents, to each urban area when the majority of the COA population falls within the urban area. Major conurbations are sub-divided if localities can be distinguished (www.ons.gov.uk). For the purpose of this study, we defined city boundaries by all Lower Layer Super Output Areas (LSOA) that best fit (i.e. 90 % area overlap) with the ONS urban areas. LSOAs have on average ~1,400 residents (range: 476 – 6,537) and were created by ONS to reflect homogenous neighbourhoods. They are the unit at which some of the data used in the subsequent analysis are disseminated. We excluded London from the analysis because of its unique administrative, social and economic place in Britain which makes it exceedingly different from other cities included in the study.

**Fig. 2:**
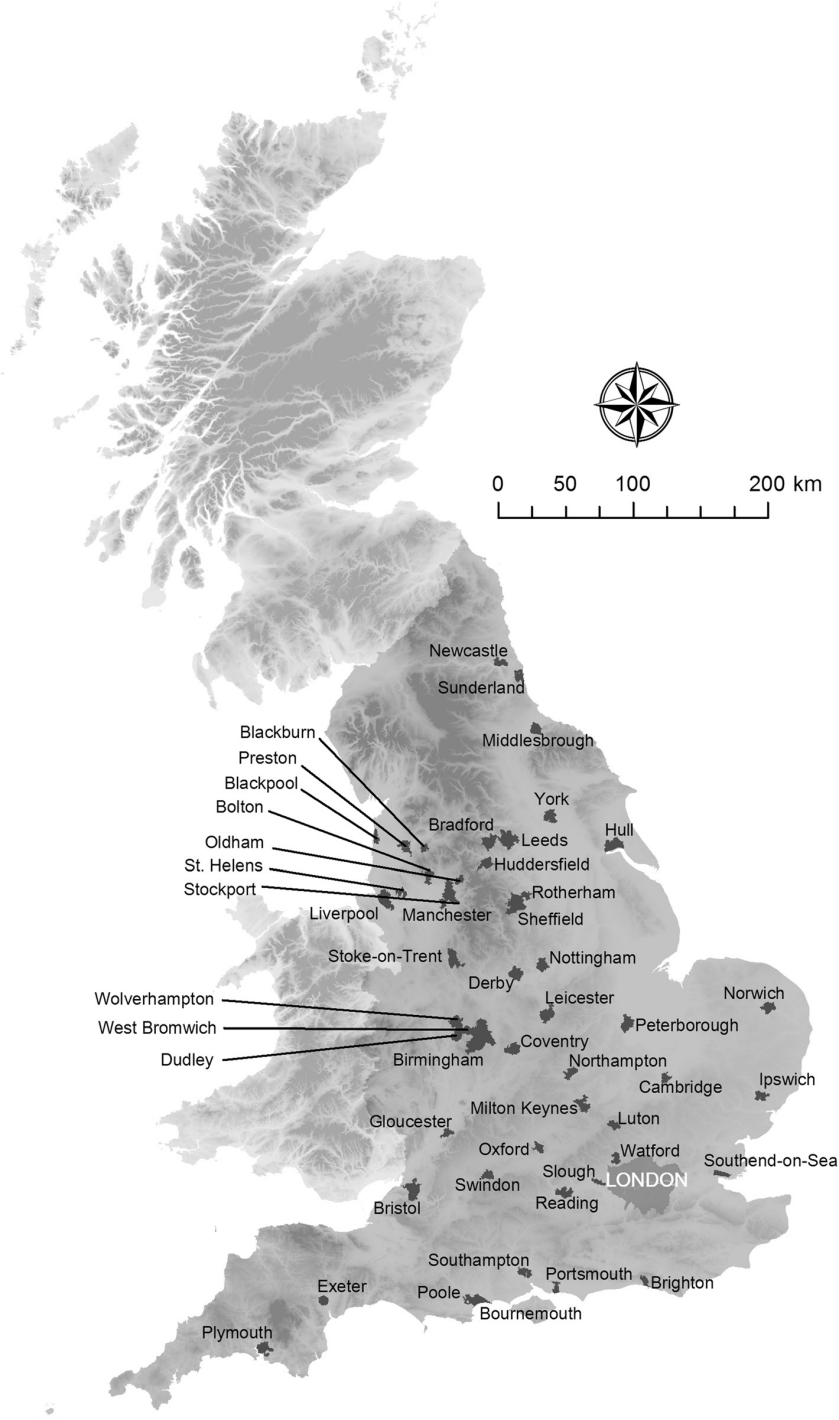
Urban area boundaries of cities in England (population ≥ 100,000 people)

Figure 2 shows the resulting urban areas included in the analysis.

### Urban metrics

To describe and characterise each of the 50 cities, we developed urban metrics within a geographic information system (GIS) which quantify the overall structure, distribution and density of urban characteristics. Using this approach, we did not qualify the urban components, for example, in terms of demographic or socioeconomic profiles, housing types, crime rates, traffic profiles but focused solely on the spatial distribution of urban characteristics. A wide variety of metrics from a range of research disciplines such as statistics, transport planning, ecology and economics have been described in the literature to quantify the spatial form, distribution and patterns of transport networks and landscape patterns [17–21]. We selected urban metrics based on their potential to assess the probable influence of population distribution, road network, land cover and geographical characteristics (e.g. terrain) on human health. Table 1 describes in detail the methods and data sources used to derive the urban metrics and indicates potential health benefits and health concerns associated with each metrics. We used ArcGIS 10 (ESRI, Redlands, CA) to compile and analyse all geographical data to derive the urban metrics.

**Table 1:** Description of urban metrics quantifying patterns of population, road network, land cover and geographical characteristics

Theme	Urban characteristic	Urban metrics	Data source	Potential health related benefits	Potential health related concerns
Population	Population density	Population density (pop/km^2^): total city population over city area in square kilometres	ONS, Census 2001	High population density: • easy to walk/cycle • increase of physical activity [15]	High population density: • spread of infectious diseases • elevated air pollution levels • potential loss of green space • social stress
Road network	Walkability of cities	Minor road density (km/km^2^): length in kilometres of minor roads and B roads over city area (km^2^)^a^	OS, Meridian 2	High minor road density: • easy to walk/cycle • increase of physical activity [15]	High minor road density: • elevated pollution levels • higher road traffic noise levels
	Connectivity of road network	Junction density (N/km^2^): number of A roads, B roads and minor road junctions over city area (km^2^)^a^	OS, Meridian 2	High junction density: • easy to walk/cycle • increase of physical activity [15]	High junction density: • elevated air pollution levels • higher road traffic noise levels • increased number of road traffic accidents related to road junctions
	Urban sprawl	Population within 100 m of major roads (%): proportion of postcode headcount population within 100 m of A roads^a^	OS, Meridian 2; ONS, Census 2001		High population density close to major roads: • higher noise and air pollution exposure across city population • less likely to walk/cycle and reduction in physical activity [27] • increased number of road traffic accidents related to higher speed
Land cover	Land cover mix	Shannon’s diversity index (SDI) [31]: $H=-{\displaystyle \sum_{i=1}^s{p}_i\kern0.2em ln\kern0.2em {p}_i\kern0.2em }$ where *p*_*i*_ is the proportion of land cover *i* relative to the total number of land cover classes	CEH, Land Cover Map 2000; EEA, CORINE Land Cover 1990 (vs 12/2000)	High land cover mix: • easy to walk/cycle • increase of physical activity [15] • less car usage • lower air pollution levels	High land cover mix: • residential areas potentially close to polluted areas
Geographical characteristics	Terrain	Altitude range (m)	OS, Land-Form PANORAMA	Hilly terrain: • increase in physical exercise if walking or cycling	Hilly terrain: • Less likely to walk/cycle

^a^Road classification in the UK is classified by the Department of Transport (DfT) as follows: A roads connect areas of regional importance, B roads connect places of local significance and minor roads are roads without classification by the DfT, mostly local roads intended for local traffic [28]

Residents living in urban areas are not only receptive to factors potentially impacting on their health; population density is also an important exposure in itself. Living in highly populated areas has been shown to be a risk factor for psychiatric diseases and anxiety disorders due to social stress [22, 23]. But high population density has also been linked to better accessibility and walkability of cities which encourages physical activity and its associated health benefits [24]. To assess population density across each city, we aggregated 2001 census population from COAs to city level and calculated the proportion of city population by the city area.

The road network influences, as well as reflects, the population distribution in a city and is a major factor of urban sprawl [25]. Road networks exert powerful influences on human behaviours and lifestyle and indirectly influence living condition (e.g. by loss of open space) and human health. They are important risk factors, for example, via accidents, noise pollution, traffic-related air pollution and a means of spread of infectious diseases [26]. To analyse the structure of the road network we followed previous literature and explored the walkability, connectivity and urban sprawl for each city [15, 27]. Urban metrics are described in detail in Table 1. We used Ordnance Survey (OS) Meridian2, a 1:50,000 scale map which differentiates between motorways, A roads, B roads and minor roads to describe the road network [28]. We defined junctions as intersections of more than 2 lines of the road network.

Land cover is a main indicator of urban form and function. It provides important, though often indirect, determinates and reflections of population distribution and human activities. Land cover mix within cities has previously been defined as an important urban characteristics affecting both car usage and consequently the air pollution levels [29] as well as the walkability and physical activity levels within cities [30]. To quantify land cover mix we used the Shannon’s Diversity Index [31], which is a measure of relative land cover diversity accounting for the abundance of different land cover classes. To obtain detailed information on land cover within each city we combined Land Cover Map (LCM) 2000 and CORINE land cover, for details see Additional file 1.

The historic urban development and spread of a city is inevitably influenced by the terrain; and topographic factors help to shape both its physical and socioeconomic characteristics. We extracted information on the altitude range for each city by overlaying the city boundaries with the Land-Form PANORAMA digital terrain model (DTM) from OS (horizontal resolution: 50 m; vertical resolution: 1 m).

### Health data

We included all registered deaths between 1^st^ January 2002 and 31^st^ December 2009. In addition to age-standardised mortality from all causes we also explored mortality for the leading specific causes of death: mortality from cardiovascular diseases (CVD) (ICD-10 codes I00-I99; ICD-9 390–459) and as a subset coronary heart disease (CHD) (ICD-10 codes I20-I25; ICD-9 410–414) and stroke (ICD-10 codes I61, I63, I64; ICD-9 434.91); and mortality from traffic accidents (ICD-10 codes V01-V89; ICD-9 E810-E829). We purposefully selected causes of mortality with different aetiologies: we included deaths from CVD as they may be influenced by physical activity levels, which are potentially influenced by the walkability of the city, the amount of green space present and the topographical layout; CVD mortality has also been linked to elevated levels of air pollution in a city. Traffic accidents are indicative of the street layout of the cities.

Annual age- sex-specific population numbers and mortality counts were extracted from databases held by the UK Small Area Health Statistics Unit (SAHSU), Imperial College London. The mortality and population data were supplied by the ONS, derived from the national mortality registrations and the Census.

### Confounders

We adjusted for socioeconomic status and smoking, two factors which are known risk factors for premature mortality, and in sensitivity analysis for traffic-related air pollution. We used the income deprivation domain from the 2004 English Index of Multiple Deprivation (IMD) to adjust for socioeconomic status [32]. This provides the proportion of people on income support within each LSOA, which we aggregated using population weights for each city. Information on smoking rates was not readily available for each city. Instead, we followed other studies and used smoothed age-sex standardised relative risks (RRs) for lung cancer mortality (ICD-10 codes C33, C34; ICD-9 162), 2002 – 2009, at the city level as a proxy measure for long-term smoking prevalence [33]. To adjust for air pollution, we used annual average nitrogen dioxide (NO_2_) concentrations for 2009 on a 200 m resolution grid which were modelled with a Land Use Regression model using information on high and low density urban, semi-natural land and length of major roads [34]. We aggregated NO_2_ estimates to city level using population weights to better represent exposure of city residents.

### Statistical analysis

We conducted separate analyses for women and men to account for gender differences previously observed in studies related to urban characteristics and mortality [35, 36]. We analysed premature mortality which we defined as deaths of those below the age of 65 to account for the influence of health-related migration of older age groups [37].

**Fig. 3:**
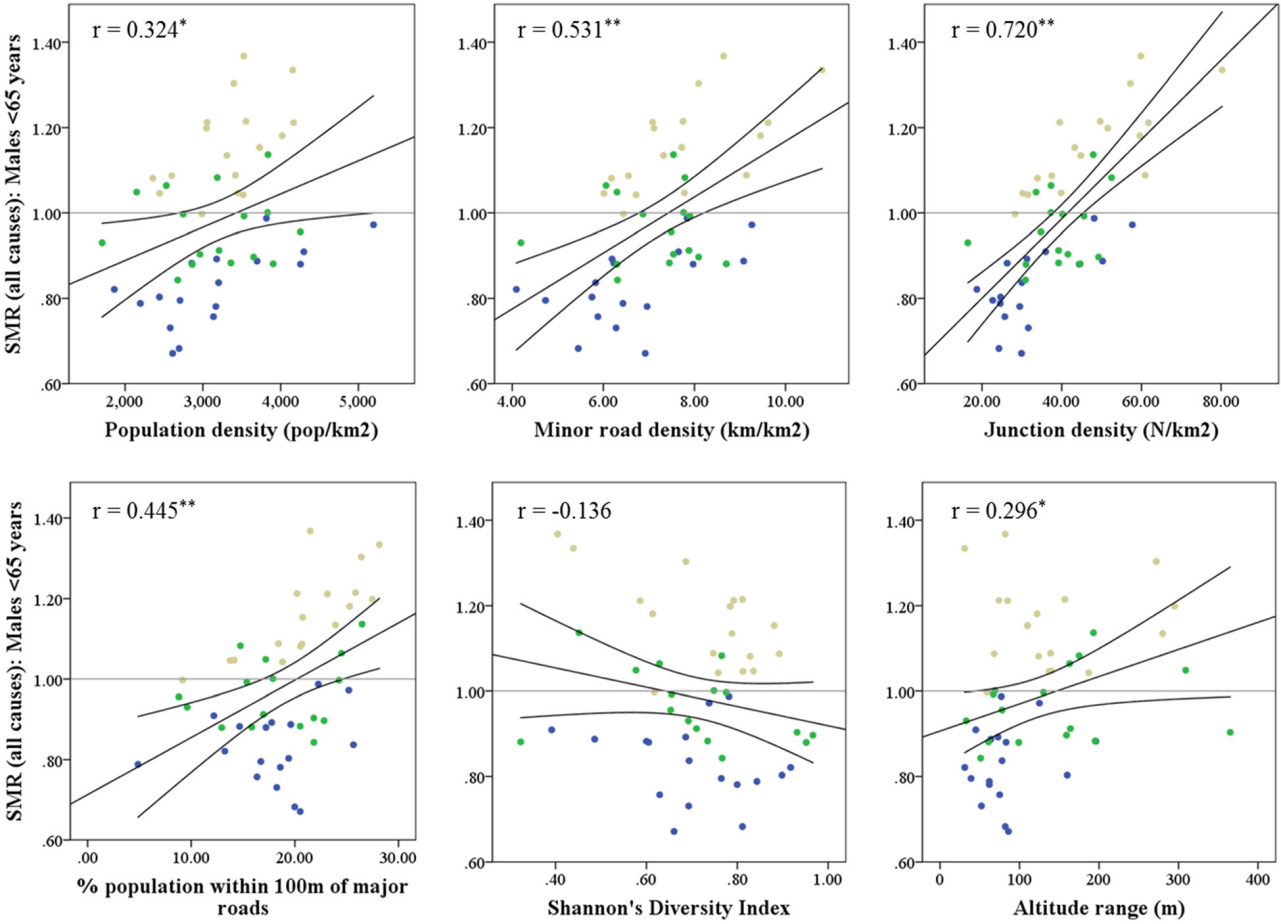
Scatterplot and correlations between Standardised Mortality Ratio (SMR) for premature mortality from all causes for males and urban metrics. Cities in the most deprived tertile are shown in beige, in the medium tertile in green and in the least deprived tertile in blue. Black line indicates line of unity with 95 % Confidence Intervals. * *p* < 0.05; ** *p* < 0.01

To calculate age-specific expected number of deaths for each city, we multiplied the population at risk, defined as the population within each city, with the mortality rate across all cities in the study. We calculated the Standardised Mortality Ratio (SMR) as the ratio between observed and expected number of deaths. To explore associations between the different urban metrics and age standardised mortality rates in cities, we used Poisson regression models. The dependent variable was the number of observed deaths in each city; the expected number was entered as the offset variable. We categorised urban metrics into tertiles because we did not assume a linear effect between mortality and urban metrics (see Fig. 3); the lowest tertile (tertile 1) was the reference group.

In sensitivity analyses we: (1) looked at deaths in all ages and older age (65 and above) and (2) adjusted the analysis in addition for traffic-related air pollution as this might be associated with the transport related metrics used here and has been previously linked to CVD mortality [38].

Statistical analysis was performed in R version 3.1.3.

### Ethics statement

The study uses SAHSU mortality data, supplied from the Office for National Statistics; data use was covered by approval from the National Research Ethics Service - reference 12/LO/0566 and 12/LO/0567 - and by Health Research Authority Confidentially Advisory Group (HRA-CAG) for Section 251 support (HRA-14/CAG/1039); suspending National Information Governance Board and Ethics and Confidentiality Committee approval (NIGB – ECC 2-06(a)/2009).

## Results

### Descriptive analysis

We saw a big contrast for urban metrics between the cities (Additional file 2). The mean population density was 3,220 people per km^2^ (Inter Quartile Range [IQR] 1,015 people/km^2^). Minor road density (mean 7.2 km/km^2^; IQR 1.6 km/km^2^) and junction density (mean 38 N/km^2^ IQR: 18 N/km^2^) were highly correlated (Pearson’s r = 0.92, Additional file 3) and were both highest in Blackpool (minor road density max 10.8 km/km^2^; junction density max 80 N/km^2^). We observed very high variability in the percentage of population living within 100 m of a major road; only 5 % of the population lived close to major roads in Milton Keynes compared to 28 % in Blackpool (mean 19 %; IQR 7 %). Based on the Shannon Diversity Index (mean 1.7; IQR 0.3), the city with the highest land cover mix was Huddersfield (SDI max 2.19) and Bournemouth, where the residential land cover class dominates, had the lowest (SDI min 1.37). The altitude range varied considerably between 7 m in Reading and 280 m in Bradford (mean 121 m; IQR 95 m).

During the study period 1^st^ January 2002 to 31^st^ December 2009 there were 200,200 premature deaths (all ages: 1,055,788 deaths) within the 50 cities included in the analysis (Female: 74,646; Male: 125,554): 24 % of those were from CVD (Female: 13,249; Male: 33,903), 14 % from CHD (Female: 5,980; Male: 22,455); 2 % from stroke (Female: 1,598; Male: 2,879) and 2 % from traffic accidents (Female: 653; Male: 2,918). City population ranged from 106,360 in Oldham to 1,076,191 in Birmingham and number of deaths varied considerably between the cities (see Table 2).

**Table 2:** Variability in observed number of deaths by city over the study period 1^st^ January 2002 to 31^st^ December 2009 (premature mortality below the age of 65 and all deaths)

Cause of mortality	City variability in observed deaths: mean (minimum and maximum)	
	Premature deaths	All deaths
All-cause	4,004 (1,429 – 17,044)	21,116 (8,583 – 99,729)
CVD	943 (261 – 4,789)	7,488 (3,004 – 35,540)
CHD	569 (143 – 2,972)	3,725 (1,388 – 17,895)
Stroke	88 (21 – 481)	1,305 (510 – 6,362)
Traffic accidents	71 (21 – 324)	89 (29 – 427)

SMRs for all deaths varied between 0.82 in Watford and 1.21 in Oldham, for deaths from CVD between 0.79 in Oxford and 1.29 in Oldham, and for deaths from traffic accidents between 0.65 in Watford and 1.75 in Peterborough (Additional file 2).

Figure 3 shows scatterplots of SMRs for premature mortality from all causes for males in relation to the urban metrics. As was expected, we saw higher SMRs for the most deprived third of cities, i.e. cities with the highest percentage of population on income support (shown in beige) compared to the most affluent third of cities (shown in blue). Apart from the Shannon Diversity Index which showed a very weak negative correlation with SMRs (r = −0.14), the other urban metrics where positively correlated with all-cause SMRs. SMRs for premature deaths in males were higher in cities with higher minor road density, junction density and percentage of the population within 100 m of major roads, with Pearson’s r = 0.53, 0.72 and 0.45, respectively. Patterns and magnitude of associations were similar for females (r = 0.50, 0.67 and 0.40 for minor road density, junction density and percentage of population within 100 m of major roads, respectively).

### Main analysis

The results from univariate and multivariate Poisson regression analyses stratified by females and males are summarised in Table 3.

**Table 3:** Association between urban metrics and premature mortality from all causes and mortality due to CVD and traffic accidents

Urban metrics	Model 1 (adjusted for age)		Model 2 (additionally adjusted for deprivation and lung cancer mortality)	
	RR (95 % CI)		RR (95 % CI)	
	Female (<65)	Male (<65)	Female (<65)	Male (<65)
	All-cause mortality			
Population density T2	1.09 [1.07 – 1.11]*	1.12 [1.10 – 1.14]*	1.02 [0.99 – 1.04]	1.01 [0.99 – 1.02]
T3	1.17 [1.15 – 1.19]*	1.19 [1.18 – 1.21]*	1.10 [1.09 – 1.13]*	1.09 [1.07 – 1.10]*
Minor Road Density T2	1.13 [1.11 – 1.15]*	1.15 [1.14 – 1.17]*	1.03 [1.00 – 1.05]*	1.01 [0.99 – 1.03]
T3	1.18 [1.16 – 1.20]*	1.21 [1.19 – 1.23]*	1.07 [1.05 – 1.09]*	1.07 [1.05 – 1.09]*
Junction Density T2	1.10 [1.08 – 1.12]*	1.09 [1.07 – 1.10]*	1.09 [1.06 – 1.11]*	1.07 [1.05 – 1.08]*
T3	1.20 [1.18 – 1.22]*	1.23 [1.21 – 1.24]*	1.12 [1.10 – 1.15]*	1.12 [1.10 – 1.14]*
% pop close to road T2	1.02 [1.00 – 1.03]	1.02 [1.01 – 1.03]*	1.01 [0.99 – 1.03]	1.00 [0.98 – 1.01]
T3	1.15 [1.13 – 1.17]*	1.15 [1.14 – 1.17]*	1.07 [1.05 – 1.09]*	1.05 [1.03 – 1.07]*
Shannon Diversity T2	1.02 [1.01 – 1.04]*	1.02 [1.01 – 1.04]*	0.99 [0.97 – 1.00]	1.00 [0.98 – 1.01]
T3	0.96 [0.94 – 0.97]*	0.95 [0.94 – 0.97]*	0.97 [0.95 – 0.99]	0.99 [0.97 – 1.00]
Altitude T2	1.09 [1.07 – 1.11]*	1.09 [1.08 – 1.11]*	1.02 [1.00 – 1.04]	1.01 [0.99 – 1.03]
T3	1.05 [1.03 – 1.07]*	1.09 [1.07 – 1.10]*	0.97 [0.95 – 0.99]*	0.98 [0.97 – 1.00]
	CVD mortality			
Population density T2	1.15 [1.10 – 1.21]*	1.17 [1.14 – 1.21]*	1.04 [0.99 – 1.09]	1.04 [1.01 – 1.07]*
T3	1.22 [1.16 – 1.27]*	1.21 [1.17 – 1.24]*	1.13 [1.07 – 1.18]*	1.10 [1.06 – 1.13]*
Minor Road Density T2	1.21 [1.16 – 1.27]*	1.21 [1.18 – 1.25]*	1.05 [1.00 – 1.11]	1.05 [1.01 – 1.09]*
T3	1.27 [1.21 – 1.33]*	1.24 [1.21 – 1.28]*	1.10 [1.05 – 1.16]*	1.09 [1.06 – 1.13]*
Junction Density T2	1.13 [1.08 – 1.18]*	1.10 [1.07 – 1.13]*	1.12 [1.07 – 1.18]*	1.09 [1.06 – 1.12]*
T3	1.23 [1.22 – 1.32]*	1.23 [1.20 – 1.26]*	1.16 [1.10 – 1.22]*	1.12 [1.09 – 1.16]*
% pop close to road T2	1.03 [0.98 – 1.07]	1.01 [0.98 – 1.04]	1.01 [0.96 – 1.06]	0.98 [0.95 – 1.01]
T3	1.17 [1.12 – 1.21]*	1.15 [1.12 – 1.18]*	1.04 [1.00 – 1.09]	1.04 [1.01 – 1.07]*
Shannon Diversity T2	1.03 [0.99 – 1.07]	1.04 [1.02 – 1.07]*	0.97 [0.93 – 1.01]	1.02 [0.99 – 1.04]
T3	0.94 [0.90 – 0.99]*	0.94 [0.91 – 0.96]*	0.95 [0.91 – 1.00]	0.97 [0.94 – 1.00]
Altitude T2	1.14 [1.09 – 1.20]*	1.10 [1.07 – 1.13]*	1.04 [0.99 – 1.09]	1.02 [0.99 – 1.05]
T3	1.11 [1.07 – 1.16]*	1.12 [1.09 – 1.15]*	1.00 [0.96 – 1.05]	1.01 [0.98 – 1.04]
	Traffic accident mortality			
Population density T2	0.90 [0.74 – 1.09]	0.92 [0.84 – 1.01]	0.90 [0.73 – 1.11]	0.91 [0.82 – 1.00]
T3	0.77 [0.63 – 0.95]*	0.79 [0.72 – 0.87]*	0.75 [0.60 – 0.93]*	0.77 [0.70 – 0.85]*
Minor Road Density T2	0.84 [0.70 – 1.02]	0.91 [0.83 – 1.00]	0.83 [0.66 – 1.05]	0.87 [0.78 – 0.97]*
T3	0.76 [0.63 – 0.93]*	0.77 [0.70 – 0.84]*	0.75 [0.60 – 0.93]*	0.71 [0.64 – 0.78]*
Junction Density T2	0.93 [0.76 – 1.13]	1.04 [0.95 – 1.14]	0.93 [0.75 – 1.15]	0.99 [0.89 – 1.10]
T3	0.93 [0.77 – 1.11]	0.91 [0.84 – 1.00]*	0.90 [0.72 – 1.13]	0.81 [0.73 – 0.91]*
% pop close to road T2	0.73 [0.60 – 0.88]*	0.95 [0.87 – 1.04]	0.68 [0.55 – 0.85]*	0.98 [0.89 – 1.09]
T3	0.86 [0.72 – 1.03]	0.89 [0.82 – 0.97]*	0.87 [0.72 – 1.05]	0.86 [0.78 – 0.95]*
Shannon Diversity T2	1.08 [0.91 – 1.30]	1.09 [1.00 – 1.19]*	1.13 [0.93 – 1.40]	1.09 [1.00 – 1.19]*
T3	1.36 [1.11 – 1.66]*	1.18 [1.07 – 1.30]*	1.39 [1.12 – 1.73]*	1.22 [1.10 – 1.35]*
Altitude T2	0.68 [0.55 – 0.83]*	0.77 [0.70 – 0.85]*	0.66 [0.54 – 0.82]*	0.74 [0.67 – 0.82]*
T3	0.79 [0.66 – 0.95]*	0.94 [0.86 – 1.03]	0.80 [0.66 – 0.98]*	0.94 [0.85 – 1.04]

*p < 0.05

We observed a statistically significant increase in risk of premature all-cause and CVD mortality with increasing population density and transport related metrics after adjustment; while the risk of traffic accident mortality decreased. Results for CHD and stroke (not shown) were similar to CVD. Adjusting for deprivation and lung cancer mortality did reduce observed risks but most remained statistically significant. For both females and males, associations were strongest after adjustment for all-cause and CVD mortality with increasing minor road and junction density: we saw a 16 % higher risk of premature CVD mortality in females across cities with the highest junction density compared to lowest third (RR 1.16, CI 1.10 – 1.22) and 12 % higher risk in males (RR 1.12, CI 1.09 – 1.16).

The decrease in deaths from traffic accidents was most strongly associated with an increase in both population and minor road density, but not junction density. After adjustment we saw a 25 % (RR 0.75, CI 0.60 – 0.93) and 23 % (RR 0.77, CI 0.70 – 0.85) decreased risk in cities with the highest population density compared to the lowest third for females and males, respectively; and a decreased risk of 25 % (RR 0.75, CI 0.60 – 0.93) and 29 % (RR 0.71, CI 0.64 – 0.78) in the highest third for minor road density for females and males, respectively.

Associations between land cover mix and all-cause and CVD mortality were very low and statistically non-significant after adjustment. For deaths from traffic accidents however the RR increased significantly in cities with the highest land cover mix (Females: RR 1.39, CI 1.12 – 1.73; Males: RR 1.22, CI 1.10 – 1.35). Results for altitude range were mostly non-significant after adjustment and inconclusive.

### Sensitivity analysis

When we included deaths from all ages most RRs were close to 1.0 (Additional file 4). Exceptions were the associations of higher junction density with higher risk of all-cause (Female: RR 1.08, CI 1.07 – 1.09; Male: RR 1.05, CI 1.04 – 1.06) and CVD (Female: RR 1.07, CI 1.06 – 1.09; Male: RR 1.05, CI 1.04 – 1.07) mortality and the associations of higher minor road density and land cover mix with higher risk of deaths from traffic accidents (Female: RR 0.86, CI 0.72 – 1.04; Male: RR 0.70, CI 0.63 – 0.77 and Female: RR 1.15, CI 0.96 – 1.38; Male: RR 1.16, CI 1.05 – 1.28, respectively). These associations were in the same direction but slightly lower compared to premature mortality. Associations were similar to those for all ages when we only included deaths in those aged 65 years and above (not shown).

**Fig. 4:**
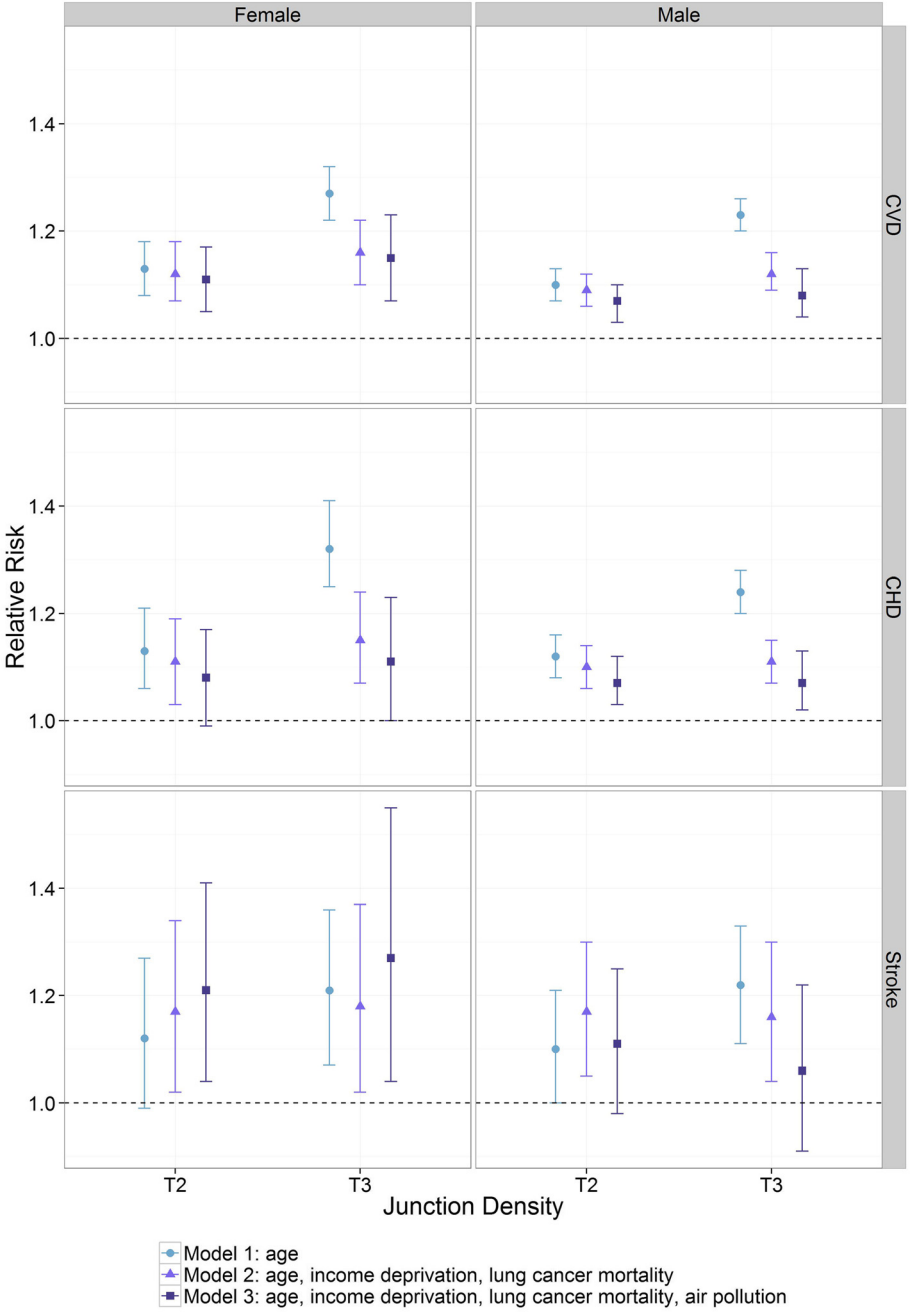
Poisson regression assessing the relationship between junction density and deaths from CVD, CHD and stroke. Shown are differences in relative risk for 2^nd^ tertile (T2) and 3^rd^ tertile (T3) in relation to the reference category (tertile 1) for Model 1: adjusting for age; Model 2: adjusting for age, income deprivation and lung cancer mortality and Model 3: adjusting as Model 2 plus NO_2_ air pollution

Figure 4 shows the effect of additional adjustment for air pollution on CVD, CHD and stroke risks and their association with junction density. We saw only a small decrease in RRs after including air pollution in our model. The RRs for deaths from stroke, however, increased for females and decreased for males after additional adjustment for air pollution. This is likely to be an artefact due to the small number of deaths.

## Discussion

Our analysis summarises between-city variations in mortality risks associated with different urban characteristics. We found that mortality risks were associated with transport network pattern and population density but associations with land cover patterns and altitude range were inconclusive. We have seen a similar increase in the risk of all-cause and CVD mortality (including deaths from CHD and stroke) with increasing population, minor road and junction density whilst the risk of death from traffic accidents decreased.

Results were of similar magnitude between all-cause and CVD mortality. CVD is one of the leading causes of death [39] and observed associations for all-cause mortality may be driven by associations between urban characteristics and CVD mortality. Fewer significant associations between urban characteristics and deaths were seen for all ages and those aged 65 years and above (see results of the sensitivity analysis, Additional file 4). These were probably similar as the majority of deaths in our study (81 %) occurred in the older age groups. Older age groups are likely to have many influences on health and this may explain why associations of mortality with urban form were less marked in this age group.

The strong associations between high density of minor roads and road junctions and the increased risk in CVD mortality were unexpected. Previous studies from the US at the neighbourhood level had reported higher attributable deaths from CHD in neighbourhoods with lower walkability, i.e. low population density, junction density and land cover mix due to reduced physical activity levels [40]. Frank et al. explored factors indicative of neighbourhood walkability for US cities and found that residential density, junction density and land use mix had a positive effect on physical active travel and body mass index [15]. Our hypothesis was similar for the UK (see Table 1), that a higher population density, road connectivity and land cover mix could be related to increased physical activity and consequently lower CVD mortality. This indicates that urban characteristics that promote walking and cycling might not be the same between the two countries.

Results from our sensitivity analysis also reject the hypothesis that increased minor road density and junction density might negatively impact on mortality risks due to increased air pollution levels. Air pollution is very likely to be on the causal pathway and one of the factors by which any underlying association between road layout and mortality is mediated. This is supported by the reduction in observed risk seen when air pollution was introduced into the analyses (see Fig. 4). Our air pollution model, however, did not account specifically for minor roads, though it did include low density urban land which contains land covered by minor roads. Also, the model did not include traffic speed variability (i.e. lower speeds close to junctions leading to generally higher air pollution (i.e. hot spots)). We may have, therefore, variably under-adjusted for air pollution related to minor roads and around road junctions.

We did see, however, statistically significant negative impact, although small, of urban sprawl, measured in our study as the percentage of population within 100 m of major roads, on all-cause mortality. This is in line with research from the US where urban sprawl, measured using a complex index, has been shown to be negatively related to body mass index, obesity, heart disease, high blood pressure and diabetes [27]. Our study did show a protective effect of reduced deaths from traffic accidents in cities with higher minor road and junction density and greater land use mix. These are factors that likely reduce the overall traffic speed and volume and consequently traffic accidents [41].

We found minor differences in associations between women and men, with women showing mildly stronger associations between urban characteristics and mortality risks for all analysed health outcomes, in particular CVD and stroke. Differential associations between women and men have previously been reported in relation to community quality and health [42]. In particular the social environment and to a lesser degree the physical environment have been shown to impact more strongly on women’s health than men’s.

This is the first study to examine the associations between urban form and mortality rates in English cities to explore if the layout of cities has an effect on the health of the urban population. Our study benefitted from the large general population sample which included all 1,055,788 registered deaths that occurred during our study period, providing sufficient statistical power to detect moderate associations. We used high-resolution geographical information to compute urban metrics to characterise the 50 cities included in the study.

To compare mortality rates and risks between the cities we used an ecological, cross-sectional study design. The study is therefore hypothesis generating but does not allow to demonstrate causality [43]. Observed associations might be subject to ecological bias and are not directly transferable to individuals within cities.

Health inequalities in Great Britain are well established [44] and have a historical continuity in geography [45]. Rates of premature mortality are generally greater in the North of England, with areas of declining industry and employment particularly affected [46]. To account for these spatial differences, we adjusted for the socioeconomic rank of the cities. Nevertheless, our analysis might be prone to residual socioeconomic confounding which could be explored in future analyses by including spatial models such as autocorrelation statistics. Residual confounding, particularly by socioeconomic status is always a potential issue in ecological studies, in particular given the strong reduction in risk estimates we have seen, after adjustment for socioeconomic variables. Further analyses with individual-level confounder information would help explore this issue and provide evidence with respect to whether associations are causal. This was not possible in this study as we used routinely collected health data which does not include this information.

Due to the use of routinely collected health and population data we did not have information on individual migration during our 8 year study period. The use of cities as units of analysis, however, might extenuate the effect of mobility often affecting small area analysis, for example at the neighbourhood level. Furthermore, we could not consider the lag period between exposure and outcome nor account for city development during the 8 year period due to lack of temporal differential exposure and confounder data.

Our unit of analysis was the city. Future research should explore associations at different spatial scales, for example, to determine if neighbourhood characteristics are more important in determining urban health than city characteristics and if this is universal for all urban metrics or if some impact more locally or even at the individual level whilst others act at a city-wide scale. A study exploring the association of city-level greenness and mortality risks, for example, found that previous results at the small area level were not directly transferable to the city level which indicates that different urban characteristics impact on health at different levels. [47].

## Conclusion

Associations between urban metrics and mortality observed in this study can highlight characteristics of urban form and structure that have negative effects on the overall health of urban communities. Future urban planning and regeneration strategies can benefit from such knowledge to promote a healthy living environment for an increasing urban population.

## Additional files

Additional file 1:
**Description of the procedure for combining Land Cover Map 2000 and CORINE land cover.** (PDF 569 kb) Additional file 2:
**City characteristics: Standardised Mortality Ratios (SMRs) for all-cause and cause specific mortality and urban metrics.** (PDF 78 kb) Additional file 3:
**Pearson’s correlation between urban metrics.** (PDF 54 kb) Additional file 4:
**Association between urban metrics and mortality from all causes and mortality due to CVD and traffic accidents.** Shown are differences in relative risk (RR) for 2^nd^ tertile (T2) and 3^rd^ tertile (T3) in relation to the reference category (1^st^ tertile). (PDF 104 kb) Additional file 5:
**Peer review reports.** (PDF 95 kb)
