# Supplementary material for: Associations between urban metrics and mortality rates in England
Source: Environ Health. 2016 Mar 8;15(Suppl 1):34. doi: 10.1186/s12940-016-0106-3 (PMC4895780; doi:10.1186/s12940-016-0106-3)
Supplement: Additional file 1: — Description of the procedure for combining Land Cover Map 2000 and CORINE land cover. (PDF 569 kb) [file 12940_2016_106_MOESM1_ESM.pdf]

Additional File 1: Description of the procedure for combining Land Cover Map 2000 and CORINE land cover

There are two main sources for land cover data in Great Britain: the Land Cover Map for Great Britain produced by the Countryside Survey, and the EU-wide CORINE Land Cover compiled by the European Environmental Agency. The national Land Cover Map 2000 (LCM2000) has been derived from LANDSAT satellites and processed by the Centre for Ecology & Hydrology to generate a 26-fold classification of land cover classes, mapped at a 25 m resolution (Fuller et al. 2002. The UK Land Cover Map 2000: construction of a parcel-based vector map from satellite images. Cartographic Journal 2002, 39: 15-25). There are, however, only two urban classes ('built-up areas, gardens' and 'continuous urban') within the LCM2000 (see Figure 1.1), limiting its use for urban areas.

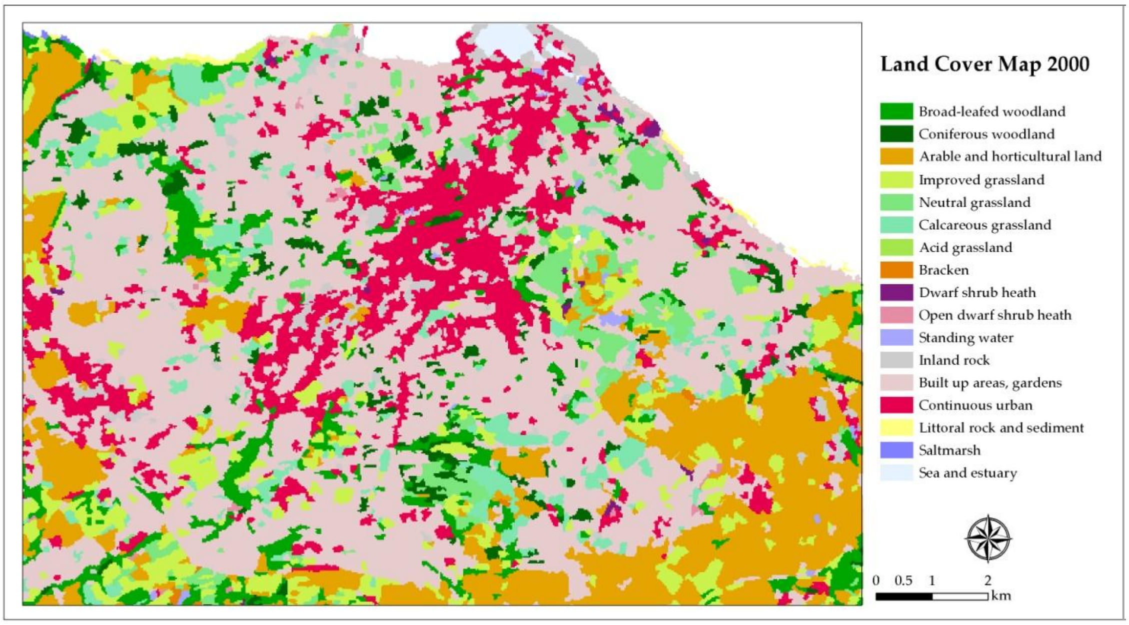

Figure 1.1 Land Cover Map 2000 for Edinburgh

The EU-wide CORINE land cover is jointly produced by the European Commission and the European member states (http://www.eea.europa.eu/publications/COR0-landcover). For its UK contribution to the CORINE land cover map, the Centre for Ecology & Hydrology generalised the more detailed LCM2000 and reclassified it according to the CORINE classification. CORINE has eleven urban classes but is only available at a 100 m resolution. As this resolution is too coarse for inter-city analysis (see Figure 1.2) the CORINE land cover cannot be used as a base.

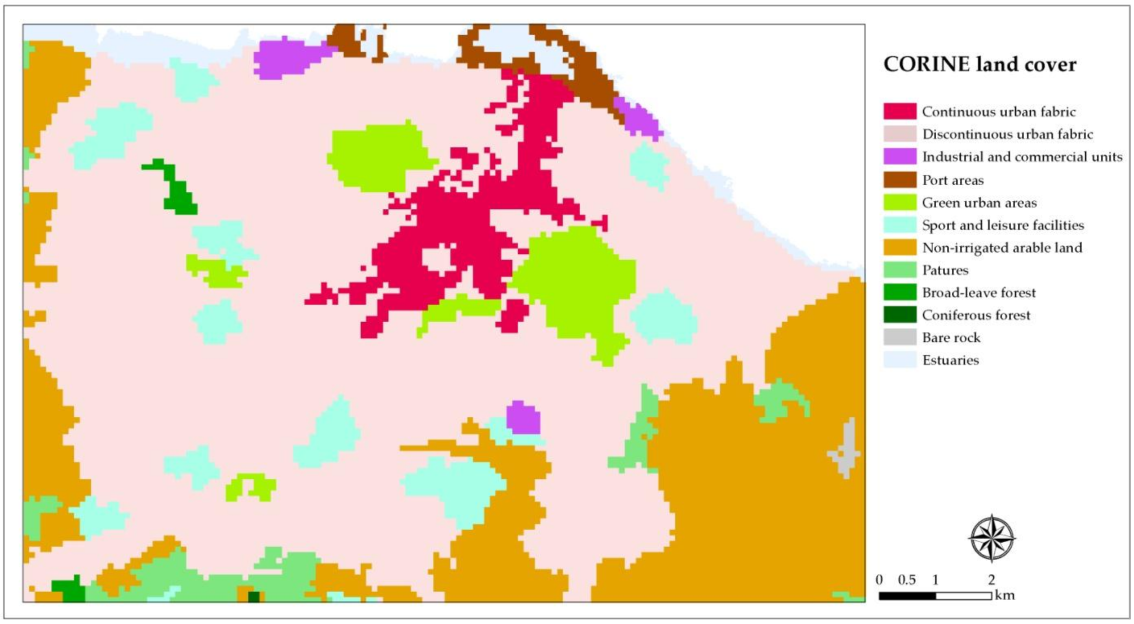

Figure 1.2 CORINE land cover for Edinburgh

To obtain detailed information on land cover within each city we therefore combined LCM 2000 and CORINE by incorporating the CORINE urban classes (e.g. industrial units, green urban areas, etc.) into the LCM 2000. We used LCM2000 as the base data and intersected LCM2000 urban areas (i.e. ‘Built up areas, gardens’, ‘Continuous urban’) with CORINE. The two main CORINE urban classes, continuous urban fabric and discontinuous urban fabric, are assumed to represent the same urban characteristics as the two urban classes in the LCM2000 and are not further considered. The CORINE urban classes of interest are the ones further subdividing the urban area, such as industrial or commercial units or green urban areas. Where these CORINE urban classes fall within the urban areas defined in LCM2000 they are used to subdivide the urban land; otherwise (i.e. where they fall outside the urban boundary indicated by LCM2000), they are ignored. All other non-urban CORINE land cover classes falling within the LCM2000 urban areas are also ignored.

Source	*Original land cover class*	*Reclassification*	*LC code*
Land Cover Map 2000	broad-leaved/mixed woodland<br>coniferous woodland	woodland	1
	arable & horticulture: cereals<br>arable & horticulture: non-cereal or unknown<br>arable & horticulture: not annual crop	arable land	2
	improved grassland<br>improved grassland: setaside grass	managed grassland	3
	neutral grassland: rough grass<br>neutral grassland: managed	unmanaged grassland	4

	calcareous grass<br>acid grass		
	bracken<br>dwarf shrub heath<br>open shrub heath	dry semi-natural	5
	fen, marsh, swamp<br>bog	wet semi-natural	6
	standing water	standing water	7
	montane habitats<br>inland bare ground<br>supra-littoral rock<br>supra-littoral sediment	rock & sediment	8
	sea/estuary<br>water (inland)<br>littoral rock	sea & marine	9
	built-up areas, gardens	built-up areas, gardens	10
	continuous urban	continuous urban	11
CORINE Land Cover	industrial or commercial units	industrial land	12
	road and rail networks	roads & rail	13
	port areas	ports	14
	airports	airports	15
	dump sites	dump sites	16
	green urban areas	green urban areas	17
	sport and leisure facilities	sport & leisure facilities	18

To simplify the land cover, classes are restructured according to their relevance to urban function and population distribution (see Table 1.1). The result is a mixture of different land cover classes from two different sources. While the combination of different data of different resolution obviously results in classification errors, in the context of this analysis - namely to compute urban metrics - these are considered to be acceptable.

The process of intersecting the urban classes of LCM2000 with the CORINE urban land cover classes, and clipping the land cover to the city boundary, inevitably creates tiny sliver polygons. These concentrate around the edges of the sample cities as well as in inner urban areas where, due to the intersection, the parcels are split into several polygons. These sliver polygons might have an effect on the resulting urban metrics and they are, therefore, excluded from these analyses. The LCM2000 defines the smallest mappable land cover unit as being greater than 0.5 ha; the smallest parcels in the CORINE land cover map have an area of 1 ha. The threshold, therefore, for patches to be included in the statistical analysis is set to 0.5 ha.
