# Supplementary material for: Associations between urban metrics and mortality rates in England
Source: Environ Health. 2016 Mar 8;15(Suppl 1):34. doi: 10.1186/s12940-016-0106-3 (PMC4895780; doi:10.1186/s12940-016-0106-3)
Supplement: Additional file 2: — City characteristics: Standardised Mortality Ratios (SMRs) for all-cause and cause specific mortality and urban metrics. (PDF 78 kb) [file 12940_2016_106_MOESM2_ESM.pdf]

Additional File 2 - City characteristics: Standardised Mortality Ratios (SMRs) for all-cause and cause specific mortality and urban metrics

City	SMRs			Urban metrics								
	All cause	CVD	Traffic Accidents	City size (km^2)	Population density (pop/km^2)	Minor road density (km/km^2)	Junction density (N/km^2)	Population within 100m of major roads (%)	Shannon Diversity Index	Altitude range (m)	Population on income support (%)	NO^2 (µg/m^3)
Birmingham	.99	1.03	1.13	305.9	3,518	6.7	31.5	19	1.72	187	24	37
Blackburn	1.19	.94	.84	26.7	3,554	7.8	49.7	26	1.71	157	26	29
Blackpool	1.14	.89	1.07	34.0	4,154	10.8	80.2	28	1.51	31	21	28
Bolton	1.13	1.15	1.32	55.1	3,047	7.1	51.6	27	1.86	295	23	33
Bournemouth	.91	1.00	1.02	44.2	3,697	9.1	50.3	20	1.37	64	13	27
Bradford	1.13	.91	1.15	84.4	3,309	7.3	44.8	24	1.82	280	26	33
Brighton	.90	1.00	.97	33.6	3,837	7.5	47.9	26	2.01	193	17	27
Bristol	.94	1.05	.99	113.2	3,652	8.1	49.2	23	1.46	159	15	28
Cambridge	.88	1.09	.92	40.1	2,707	4.7	22.7	17	1.98	39	8	24
Coventry	.97	.97	.90	75.8	3,832	7.8	37.3	18	1.68	69	18	27
Derby	.92	1.15	.97	78.5	2,863	6.3	31.0	13	1.56	99	18	27
Dudley	1.01	1.03	1.03	58.0	3,361	7.5	39.2	21	1.53	195	16	36
Exeter	.89	.93	1.49	43.9	2,442	5.8	24.7	19	1.86	160	12	24
Gloucester	.91	1.06	.70	40.4	2,856	6.2	26.3	15	1.80	196	13	24
Huddersfield	1.06	.90	1.43	61.2	2,149	6.3	33.6	17	2.19	309	18	26
Hull	1.04	.88	1.15	99.4	2,987	6.4	28.3	9	1.67	59	22	26
Ipswich	.87	1.02	1.07	53.4	2,580	6.3	31.6	18	1.88	52	13	25
Leeds	1.01	1.29	.85	135.2	3,185	7.8	52.5	15	1.78	175	19	32
Leicester	1.02	.79	.80	92.3	3,529	7.9	45.6	15	1.64	67	20	29
Liverpool	1.19	1.03	1.75	109.8	4,166	9.6	61.8	23	1.44	85	30	34
Luton	1.01	1.02	.71	43.4	4,253	7.5	34.8	9	1.54	78	16	27
Manchester	1.14	.82	.83	110.7	3,526	8.6	59.9	21	1.68	82	29	38
Middlesbrough	1.07	1.01	.66	59.0	2,443	6.0	30.2	14	1.97	137	26	25
Milton Keynes	.94	1.06	1.08	80.2	2,196	6.4	24.6	5	2.01	62	11	23
Newcastle	1.10	.81	.90	48.5	4,020	9.5	59.6	25	1.57	122	26	34
Northampton	.93	1.17	.96	57.1	3,177	6.2	31.3	18	2.04	73	12	25
Norwich	.84	.97	.90	62.8	2,676	6.3	30.9	22	1.74	51	15	26
Nottingham	1.07	.93	1.32	66.0	3,729	7.7	43.2	21	1.44	110	25	31
Oldham	1.21	.89	.79	31.3	3,398	8.1	57.3	26	1.86	272	27	34
Oxford	.84	.98	1.03	42.5	3,202	5.8	30.	26	1.91	78	10	22
Peterborough	1.00	1.11	.80	80.3	1,705	4.2	16.4	10	2.05	33	17	23
Plymouth	.96	1.15	.98	75.1	3,209	7.9	39.2	17	1.60	164	16	26

Poole	.84	1.07	1.42	54.2	2,611	6.9	29.9	21	1.51	86	9	26
Portsmouth	.96	1.06	.78	35.1	5,194	9.3	57.7	25	1.73	125	14	29
Preston	1.04	.88	.99	63.0	2,746	6.9	40.2	24	1.78	130	15	27
Reading	.83	.80	.65	72.9	3,136	5.9	25.7	16	1.77	75	7	27
Rotherham	1.13	1.21	1.12	34.5	2,356	6.2	33.9	21	1.84	124	25	29
Sheffield	.98	.99	1.18	147.3	2,961	7.5	41.6	22	1.81	365	19	29
Slough	.89	.91	.74	28.3	4,299	7.7	35.9	12	1.45	45	13	32
Southampton	.89	1.03	1.13	53.6	4,255	8.0	44.4	17	1.48	83	14	29
Southend-on-Sea	.98	.94	.84	41.0	3,905	8.7	44.6	16	1.58	61	16	27
St. Helens	1.13	.89	1.07	37.1	2,600	6.6	37.5	21	1.94	68	21	30
Stockport	1.05	1.15	1.32	35.7	3,817	7.8	48.1	22	1.73	77	14	34
Stoke-on-Trent	1.09	1.00	1.02	96.5	2,529	6.1	37.3	25	1.70	163	19	24
Sunderland	1.11	.91	1.15	51.5	3,419	9.1	60.9	18	1.93	139	23	28
Swindon	.89	1.00	.97	48.3	3,167	7.0	29.5	19	1.75	62	10	22
Watford	.82	1.05	.99	42.2	2,694	5.5	24.2	20	1.84	82	8	33
West Bromwich	1.18	1.09	.92	38.8	3,054	7.1	39.5	20	1.42	74	25	41
Wolverhampton	.99	.97	.90	71.3	3,448	7.8	39.9	14	1.42	140	21	34
York	.86	1.15	.97	73.9	1,861	4.1	18.7	13	2.09	31	11	22
