# Supplementary material for: Associations between urban metrics and mortality rates in England
Source: Environ Health. 2016 Mar 8;15(Suppl 1):34. doi: 10.1186/s12940-016-0106-3 (PMC4895780; doi:10.1186/s12940-016-0106-3)
Supplement: Additional file 3: — Pearson’s correlation between urban metrics. (PDF 54 kb) [file 12940_2016_106_MOESM3_ESM.pdf]

Additional File 3: Pearson’s correlation between urban metrics

	Minor Road Density	Junction Density	% pop close to road	Shannon Diversity	Altitude
Population Density	0.803**	0.680**	0.310*	-0.606**	-0.050
Minor Road Density		0.915**	0.403**	-0.598**	0.092
Junction Density			0.593**	-0.400*	0.200
% pop close to road				-0.092	0.355*
Shannon Diversity					0.200

* p < 0.05; ** p < 0.001
