# Supplementary material for: Associations between urban metrics and mortality rates in England
Source: Environ Health. 2016 Mar 8;15(Suppl 1):34. doi: 10.1186/s12940-016-0106-3 (PMC4895780; doi:10.1186/s12940-016-0106-3)
Supplement: Additional file 4: — Association between urban metrics and mortality from all causes and mortality due to CVD and traffic accidents. Shown are differences in relative risk (RR) for 2nd tertile (T2) and 3rd tertile (T3) in relation to the reference category (1st tertile). (PDF 104 kb) [file 12940_2016_106_MOESM4_ESM.pdf]

Additional File 4: Association between urban metrics and mortality from all causes and mortality due to CVD and traffic accidents.

Shown are differences in relative risk (RR) for 2nd tertile (T2) and 3rd tertile (T3) in relation to the reference category (1st tertile)

**Urban metrics**	**Model 1** (adjusted for age) **RR (95% CI)**		**Model 2** (additionally adjusted for deprivation and lung cancer mortality) **RR (95% CI)**	
	**Female (all ages)**	**Male (all ages)**	**Female (all ages)**	**Male (all ages)**
	**All-cause mortality**			
Population density T2	1.04 [1.04 – 1.05]*	1.06 [1.06 – 1.07]*	0.97 [0.97 – 0.98]*	0.98 [0.97 – 0.99]*
T3	1.06 [1.06 – 1.07]*	1.09 [1.09 – 1.10]*	1.01 [1.01 – 1.02]*	1.02 [1.01 – 1.03]*
Minor Road Density T2	1.08 [1.07 – 1.08]*	1.09 [1.08 – 1.10]*	0.98 [0.97 – 0.99]*	1.00 [0.99 – 1.01]
T3	1.09 [1.08 – 1.10]*	1.12 [1.11 – 1.23]*	1.01 [1.00 – 1.02]*	1.01 [1.00 – 1.02]*
Junction Density T2	1.08 [1.07 – 1.08]*	1.07 [1.06 – 1.08]*	1.07 [1.06 – 1.07]*	1.05 [1.04 – 1.06]*
T3	1.13 [1.12 – 1.13]*	1.14 [1.13 – 1.15]*	1.08 [1.07 – 1.09]*	1.05 [1.04 – 1.06]*
% pop close to road T2	0.98 [0.98 – 0.99]*	1.01 [1.01 – 1.02]*	0.98 [0.97 – 0.99]*	1.02 [1.01 – 1.03]*
T3	1.07 [1.06 – 1.07]*	1.10 [1.09 – 1.10]*	0.90 [1.01 – 1.02]	1.03 [1.02 – 1.04]*
Shannon Diversity T2	0.99 [0.98 – 0.99]*	1.01 [1.00 – 1.02]*	0.96 [0.96 – 0.97]*	0.98 [0.98 – 0.99]*
T3	0.98 [0.97 – 0.98]*	0.98 [0.97 – 0.99]*	0.99 [0.99 – 1.00]	0.99 [0.98 – 1.00]*
Altitude T2	1.04 [1.03 – 1.05]*	1.06 [1.05 – 1.06]*	1.00 [1.99 – 1.00]	1.02 [1.01 – 1.03]*
T3	1.03 [1.02 – 1.03]*	1.06 [1.05 – 1.07]*	0.97 [0.97 – 0.98]*	1.00 [0.99 – 1.00]
	**CVD mortality**			
Population density T2	1.01 [1.00 – 1.02]	1.07 [1.05 – 1.08]*	0.95 [0.94 – 0.96]*	0.99 [0.98 – 1.00]
T3	1.02 [1.01 – 1.03]*	1.08 [1.06 – 1.09]*	0.97 [0.96 – 0.98]*	1.01 [0.99 – 1.02]
Minor Road Density T2	1.05 [1.03 – 1.06]*	1.09 [1.08 – 1.11]*	0.97 [0.95 – 0.98]*	1.00 [0.99 – 1.01]
T3	1.06 [1.04 – 1.07]*	1.11 [1.09 – 1.12]*	0.98 [0.97 – 0.99]*	1.00 [0.99 – 1.01]
Junction Density T2	1.08 [1.07 – 1.09]*	1.08 [1.06 – 1.09]*	1.07 [1.06 – 1.09]*	1.05 [1.05 – 1.06]*
T3	1.11 [1.10 – 1.12]*	1.14 [1.13 – 1.15]*	1.07 [1.06 – 1.09]*	1.05 [1.04 – 1.07]*
% pop close to road T2	0.97 [0.96 – 0.98]*	1.00 [0.98 – 1.01]	0.97 [0.96 – 0.98]*	1.00 [0.99 – 1.01]

	T3	1.05 [1.04 – 1.06]*	1.08 [1.07 – 1.09]*	1.01 [1.00 – 1.03]*	1.02 [1.01 – 1.03]*
Shannon Diversity	T2	0.99 [0.98 – 1.00]	1.02 [1.01 – 1.04]*	0.97 [0.96 – 0.98]*	1.00 [0.99 – 1.01]
	T3	1.00 [0.99 – 1.02]	0.99 [0.98 – 1.00]	1.03 [1.02 – 1.05]*	1.01 [0.99 – 1.02]
Altitude	T2	1.02 [1.01 – 1.04]*	1.04 [1.03 – 1.05]*	0.99 [0.98 – 1.01]	0.99 [0.98 – 1.00]
	T3	1.03 [1.02 – 1.04]*	1.07 [1.05 – 1.08]*	0.99 [0.98 – 1.00]	0.99 [0.98 – 1.01]
**Traffic accident mortality**					
Population density	T2	0.90 [0.77 – 1.05]	0.96 [0.88 – 1.05]	0.90 [0.76 – 1.07]	0.91 [0.83 – 1.00]
	T3	0.87 [0.74 – 1.02]	0.84 [0.77 – 0.92]*	0.88 [0.75 – 1.05]	0.80 [0.72 – 0.88]*
Minor Road Density	T2	0.87 [0.74 – 1.02]	0.98 [0.90 – 1.06]	0.85 [0.71 – 1.02]	0.89 [0.80 – 0.99]*
	T3	0.85 [0.73 – 1.00]*	0.81 [0.74 – 0.88]*	0.86 [0.72 – 1.04]	0.70 [0.63 – 0.77]*
Junction Density	T2	0.91 [0.79 – 1.08]	1.03 [0.95 – 1.12]	0.92 [0.77 – 1.10]	0.97 [0.88 – 1.07]
	T3	0.96 [0.83 – 1.11]	0.93 [0.86 – 1.01]	0.95 [0.80 – 1.13]	0.83 [0.75 – 0.92]*
% pop close to road	T2	0.79 [0.67 – 0.92]*	1.00 [0.92 – 1.09]	0.74 [0.63 – 0.88]*	1.02 [0.93 – 1.12]
	T3	0.95 [0.82 – 1.10]	0.96 [0.89 – 1.04]	0.96 [0.82 – 1.12]	0.93 [0.86 – 1.02]
Shannon Diversity	T2	1.09 [0.95 – 1.26]	1.11 [1.03 – 1.20]*	1.10 [0.95 – 1.27]	1.11 [1.03 – 1.20]*
	T3	1.18 [1.00 – 1.40]	1.13 [1.03 – 1.24]*	1.15 [0.96 – 1.38]	1.16 [1.05 – 1.28]*
Altitude	T2	0.82 [0.70 – 0.97]*	0.81 [0.74 – 0.89]*	0.80 [0.68 – 0.95]*	0.80 [0.73 – 0.87]*
	T3	0.84 [0.73 – 0.98]*	0.98 [0.90 – 1.06]	0.84 [0.72 – 0.99]*	0.96 [0.88 – 1.05]
