# Supplementary material for: Associations between urban metrics and mortality rates in England
Source: Environ Health. 2016 Mar 8;15(Suppl 1):34. doi: 10.1186/s12940-016-0106-3 (PMC4895780; doi:10.1186/s12940-016-0106-3)
Supplement: Additional file 5: — Peer review reports. (PDF 95 kb) [file 12940_2016_106_MOESM5_ESM.pdf]

Reviewer 1

Thanks Sotiris,

Here are my comments:

The topic of the paper is interesting and there are few studies examining this. However I have some reservations regarding the analyses. To me there appears to be a high probably that most of the observed results could be explained by residual confounding. After adjusting for a limited number of potential confounders, most of the risk estimates are reduced by half. Given that the area level SES indicator may account for about half of the individual SES, there is a real possibility that with a better (individual level) SES indicator the elevated risks may disappear (A sentence in the discussion is not sufficient to address this). Furthermore I think in the models there should be adjustment for ethnicity and probably size of the city (possible in terms both of overall population size and surface area). I think compared a city of 100K people and 1M people is not easy to justify.

I am a bit confused as generally the paper refers to between city variability and premature deaths (<65) but then at times within city variability and older people (e.g. discussion p21 2U, p22 9D) are mentioned. Is focusing on <65 years old not a real limitation?

Title: I think urban form is not exactly what is examined more urban metrics

Figure 1: I find the figure confusing and it is does not take into account the more hierarchical frame that exists (e.g. urban structure affects pollution and then affects health). I think the generally used WHO determinants of disease model may be more appropriate.

Table 1 Is minor road density really a good measure of walkability? Why is green space not included? The resolution of CORINE is not great and should be mentioned. What would happen with better maps?

Results: start with description of city variables and then health variables.

Figure 3 switch around the axes. Exposures are generally on the x-axis and health outcomes on the y axis

Page 17 start the traffic accident section as a new paragraph to make it easier to read

Best wishes,

Mark

Mark J Nieuwenhuijsen PhD
Research Professor in Environmental Epidemiology
Center for Research in Environmental Epidemiology (CREAL)

Reviewer 2

Review comments on manuscript "Associations between urban form and mortality rates in England"

The authors have compared various metrics characterizing 50 major urban areas in the UK (junction density, minor road density) with mortality rates in these cities. The study uses a cross-sectional design. Associations were found between especially minor road density, junction density and all-cause and cardiovascular mortality (positive) and traffic accidents mortality (negative). The study addresses an important issue in the field of healthy urban living, as is explained well in the Introduction of the paper. The study has been conducted well and the paper is generally well written. The limitations of the study are discussed by the authors, but could be expanded a bit.

Main comments

1. A limitation of the study is the focus on city-level, as identified by the authors. Finally 50 units of observation are used, much lower than studies evaluating neighbourhood level. Provide some information about the variation of mortality risks between versus within cities, perhaps by referring to previous work.
2. The authors only evaluated individual single urban metric models. Quite of the metrics will likely be correlated, which would be useful to add to the paper. The authors should further consider two urban metric models, e.g. minor road and junction density in one model or motivate why they did not include these models.
3. The authors claim that risks for females are higher than for males. I do not think the data support such a claim, Table 3 model 2 shows basically identical risks for all-cause mortality (e.g. junction and minor road density, adjusted). CVD risks are only very mildly higher. No formal test was shown for differences between the sexes. I recommend to take out the claim from abstract and concluding sections and only flag the topic in the discussion.
4. The impact of adjustment for lung cancer rate and the socio-economic variable is impressive (less so for accidents), raising the possibility of

residual confounding. Please add to this to the brief general mentioning of residual confounding on p24. On p12 expand the single lines on lung cancer Relative risks (for what, increment) and air pollution (add LUR with indication of which variables went into the model).

5. Are the authors willing to make specific recommendations based upon their work or is further work e.g. at neighbourhood level needed?

Minor comments

1. Provide a label in the tables explaining that T2= 2d tertile, T3=third tertile, which are compared to the first tertile.
2. Table 2: number for CVD seems wrong. Why pick 2.5 and 97.5 percentile, which are min and max for n=50 observations?
3. Figure 3: consider reversing X and Y axes
4. Table 3: T3 and traffic accident should also have a *
5. P22: note that the US walkability study was on the neighborhood scale.

Reviewer 3

Associations between urban form and mortality rates in England

General comments
This is an interesting study of city-level mortality and its associations with urban morphology. It develops and brings together an array of city measures including walkability/connectivity, land cover mix and altitude range. An appropriate method (Poisson regression) is applied to estimate associations between these measures and mortality due to all causes, and several specific causes. The authors find that transport network measures (e.g. junction density) in particular were associated with mortality, and associations in general persisted after adjustment for socio-economic deprivation and lung cancer mortality (used as an indicator of smoking prevalence).

In general, the paper has merit and is a useful addition to the literature on urban health, an increasingly important issue. The methods are appropriate for the research question, and capitalise on (largely) readily available secondary data. The focus on premature mortality is justifiable, and the authors carry out sensitivity analyses to check findings against those for deaths at all ages. As the authors acknowledge, the ecological cross-sectional design has a number of limitations and cannot be used to infer causality. However, it provides a useful insight into the city's population health, and has the advantages of being near-comprehensive given the coverage of mortality registration data.

Major compulsory revisions

1. A key issue for me is that more emphasis is needed that this is a study of cities – at the city level. As the authors acknowledge, the question at hand could feasibly be addressed using smaller area data within the cities, using the same data sets aggregated to e.g. LSOAs, giving a more nuanced analysis allowing for within-city variability in exposures and outcomes. However, there is merit in the city-scale, and in general the authors do frame the study in this way, but this could be brought out more, and the value of working at this scale needs to be justified more. For example, this could be argued in the specification of the research question (end of introduction, p6). The authors do discuss this issue briefly in the penultimate paragraph of the discussion, but I think more needs to be made of this. What are the advantages of analysis at this scale? Does it have different implications to analysis at smaller scale? If the previous work cited has demonstrated that small area findings do not necessarily translate to city-level, is either of the approaches 'right' or 'wrong' for different applications? Should we expect to see associations at this scale?

2. Methods/Discussion of limitations: I'd like to see more justification of the use of tertiles to classify the exposure variables of interest. I agree that it's good to look for non-linearity, but tertiles result in a very coarse reduction of the nice continuous variables that have been carefully construed and constructed here. It's been argued recently that this approach, with specific reference to built environment variables, can result in significant loss of data and lack of comparability with other studies (Lamb & White 2015. Categorisation of built environment characteristics: the trouble with tertiles. IJBNPA 12, 19.). Could models using continuous exposure terms be assessed, even if presented as supplementary material?

Minor essential revisions

3. Abstract, Results: I don't agree that 'Associations between mortality and population patterns were weaker' (than with junction density) – the effect sizes are very similar, it's just that with population density there are opposite associations with CVD and traffic accident mortality? [see also comment 9 below]

4. P5: I think the value of Figure 1 is arguable. I appreciate the work that's gone into this, but I think it duplicates existing frameworks for the determinants of health, not least Barton &

Grant's 'Health Map', based on Dahlgren & Whitehead's work (http://www1.uwe.ac.uk/et/research/who/resourcesandtools/thehealthmap.aspx). One version is explicitly focused on 'the determinants of health and wellbeing in our cities', covering many of the same factors. I think the concentric diagram also has the advantage of indicating the interconnectedness of the different factors operating at different levels, which appear independent in Fig 1. It's nice to have a diagram, but I don't think it's worth reinventing the wheel here. I appreciate it's not necessarily desirable to reproduce existing graphics, but the Health Map is at least available under a creative commons licence? Alternatively I'd just suggest excluding the figure.

5. P10: "…motorways, A roads, B roads and minor roads…" – given the international readership of the journal, I'd suggest adding very brief definition/distinction of these UK road types?

6. P11: It's not entirely clear to me how the LCM and CORINE land cover data have been integrated; could this description be expanded a little? If CORINE is a generalisation of the LCM, how does it add value when combined back with the LCM? And what is the impact of the different minimum land parcel unit areas included in the two datasets (I think LCM is 0.5ha but CORINE is 25ha?)

7. Discussion: I understand why London was excluded, it usually does behave differently from other cities in the UK. However, it seems a shame to exclude it completely given the population and hence volume of data excluded. Are there any indications of how the measures differ for London? Is there any indication of how inclusion might have impacted on the findings?

8. Discussion: Related to comment 1, I think it would be worth reflecting on the potential for variation in the scale of processes underpinning associations between the urban form measures and mortality. The mechanisms are already discussed, by which these city characteristics might impact on health, and it would be useful to discuss the scale issue with regard to these. For example, we might expect population density and intersection density to be good indicators that are approximately representative of city-wide characteristics such as air quality, infectious disease transmission and social stress; but land cover diversity may only be more relevant on a more immediate locality scale to residential neighbourhoods, e.g. in terms of supporting physical activity.

9. Discussion, para 1: I don't agree that associations with population density and land cover patterns were "inconclusive"; they just seem to vary by health outcome, and the authors already discuss why this might be.

Discretionary revisions

10. P16 Figure 3: It's debatable, but convention is to plot outcomes on y axes and exposures on x axes; I'd find it easier to read these graphs if axes were switched.
